# Development and validation of a mathematical model of heart rate response to fluid perturbation

**DOI:** 10.1038/s41598-022-25891-y

**Published:** 2022-12-12

**Authors:** Varun Kanal, Pras Pathmanathan, Jin-Oh Hahn, George Kramer, Christopher Scully, Ramin Bighamian

**Affiliations:** 1 Office of Science and Engineering Laboratories, Center for Devices and Radiological Health, United States Food and Drug Administration, Silver Spring, MD USA; 2 Department of Mechanical Engineering, University of Maryland, College Park, MD USA; 3 Department of Anesthesiology, The University of Texas Medical Branch, Galveston, TX USA

**Keywords:** Computational models, Machine learning

## Abstract

Physiological closed-loop controlled (PCLC) medical devices monitor and automatically adjust the patient’s condition by using physiological variables as feedback, ideally with minimal human intervention to achieve the target levels set by a clinician. PCLC devices present a challenge when it comes to evaluating their performance, where conducting large clinical trials can be expensive. Virtual physiological patients simulated by validated mathematical models can be utilized to obtain pre-clinical evidence of safety and assess the performance of the PCLC medical device during normal and worst-case conditions that are unlikely to happen in a limited clinical trial. A physiological variable that plays a major role during fluid resuscitation is heart rate (HR). For in silico assessment of PCLC medical devices regarding fluid perturbation, there is currently no mathematical model of HR validated in terms of its predictive capability performance. This paper develops and validates a mathematical model of HR response using data collected from sheep subjects undergoing hemorrhage and fluid infusion. The model proved to be accurate in estimating the HR response to fluid perturbation, where averaged between 21 calibration datasets, the fitting performance showed a normalized root mean square error (NRMSE) of $7.41 \pm 2.8 \%$. The model was also evaluated in terms of model predictive capability performance via a leave-one-out procedure (21 subjects) and an independent validation dataset (6 subjects). Two different virtual cohort generation tools were used in each validation analysis. The generated envelope of virtual subjects robustly met the defined acceptance criteria, in which $95\%$ of the testing datasets presented simulated HR patterns that were within a deviation of 50% from the observed data. In addition, out of 16000 and 18522 simulated subjects for the leave-one-out and independent datasets, the model was able to generate at least one virtual subject that was close to the real subject within an error margin of $9.56 \pm 3.15\%$ and $11.1 \pm 1.22\%$ NRMSE, respectively. In conclusion, the model can generate valid virtual HR physiological responses to fluid perturbation and be incorporated into future non-clinical simulated testing setups for assessing PCLC devices intended for fluid resuscitation.

## Introduction

Physiological closed-loop controlled (PCLC) medical devices refer to technologies that use physiological variables measured from a patient as feedback in a closed loop manner to achieve the treatment objectives set by clinical staff. These types of devices are rapidly evolving and under development for multiple medical applications^1–8^. These devices use physiological feedback to make automatic adjustments to the therapy delivered to a patient that may normally be made by a clinician or other caregivers. This type of device, if well designed, can ensure timely and effective therapy while reducing side effects^9,10^. In some instances they may help shorten the length of stay in the hospital and relieve the workload of hospital staff^10,11^. A workshop conducted by the U.S. Food and Drug Administration (FDA) in 2015 discussed the importance of these types of devices and emphasized the above points^12,13^.

The use of some PCLC devices can be directly related to the patients’ survival, and failure to perform as intended can lead to deterioration of a patient’s physiological condition and, in some cases, fatal consequences^14^. Thus, a strict evaluation of a PCLC performance is essential. Traditionally, medical devices are evaluated by conducting a clinical study on humans or animals. Although a large clinical study may allow us to comprehensively evaluate the PCLC medical devices, it can take many years, be expensive or even impractical due to ethical reasons^15^. In addition, a limited clinical study may not capture all inter- and intra-subject variability during normal and worst-case physiological states that can possibly affect the performance of PCLC devices^15^. To complement the limited clinical trials and reduce the need for large clinical studies, simulated subjects with diverse physiological conditions within the intended use of the device can be used to assess the effectiveness of PCLC devices^15–17^. These virtual subjects can be created using a mathematical model that maps the treatment input to the physiological variables with which the PCLC devices operate^7,18^. These models can be generated using data collected during a smaller human or animal study^15,19,20^.

One medical application where PCLC devices can be used is fluid resuscitation after a hemorrhagic shock event, where they control the rate of fluid that is infused in hypovolemic patients. A common physiological variable that needs to be monitored during fluid resuscitation is heart rate (HR), where a PCLC device may control HR-driven physiological variables (e.g., cardiac output and blood pressure) while replacing lost fluids in patients with hypovolemia. Therefore, a mathematical model is needed that can represent the changes in HR due to fluid perturbation. Virtual subjects created from such a model can then be used as part of a larger testing platform to assess the PCLC medical devices used for fluid resuscitation.

Efforts have been made to model the cardiovascular system with HR response under different physiological scenarios^21–24^. These models are mostly high-order mathematical models that capture the underlying states of the cardiovascular response and physiological variables. Although these models are physiologically useful, they are created using data collected in a highly controlled environment through laboratory procedures that may crucially differ from the standard of care. In addition, these models are not validated in terms of their predictive capability performance and are mostly limited to a few minutes of forecasting^25–27^. Many of the HR models are also based on the sympathetic and parasympathetic branches of autonomic nervous system, which are not fully understood under the fluid perturbation scenarios and their validation requires to address the long-lasting challenge for discrimination of the dynamics of these two separate branches^26,27^. In order to simulate valid physiological HR responses to fluid perturbation with no or limited knowledge about the autonomic nervous system activity and discriminating sympathetic and parasympathetic branches, a mathematical model is preferred to be low-order to be fully adapted to each individual while generating HR response based on the inputted rates of hemorrhage and infusion. A low-order mathematical model can in turn be used with less complex and at the same time more efficient virtual cohort generation tools, leading to more suitable methods for simulating diverse physiological HR responses. To the best of our knowledge, there are no systems-level or low-order lumped parameter models of HR response to fluid perturbation.

To address the need for a model of HR response to fluid perturbation, this research presents a low-order mathematical model of HR that can be used to create virtual cohorts of HR response under hemorrhage and fluid infusion. This model can be used as part of a larger testing platform to evaluate the performance of PCLC medical devices. The proposed model has a proper trade-off between model complexity and fitting performance. For the first time, we also present numerous tests performed on the HR model validity and its predictive capability performance. Tests were performed to examine the robustness of the model by assessing its subject-specific prediction under transient and steady-state responses to hemorrhage and infusion. In addition, the model’s ability to generate fully virtual HR responses was evaluated using a leave-one-out cross-validation approach and also by testing against an independent validation dataset. To effectively assess the model’s predictive capability performance, which includes the mixed effects from the model and the virtual cohort generation tool, we employed a uniform distribution method and also developed a novel compartment method for generating virtual subjects. Applying these tools separately ensured an appropriate separation between the effects from the model and those of virtual cohort generation tool on the predictive capability performance.

The paper is structured as follows. The “Methods” section introduces the model development and numerous tests performed for model verification and validation. The “Results” section presents the outcome of the tests performed to evaluate the model. Finally, the implications of these results are presented in the “Discussion” section.

## Methods

In this section, we present the model development and validation approaches and the experimental data we used for the model evaluation. The mathematical model is designed to replicate both transient and long-term effects of fluid perturbation. Furthermore, the model is assessed in terms of its predictive capability performance through a subject-specific HR response prediction, full-scale predictive cohorts through a leave-one-out procedure as well as using an independent validation dataset.

### Model development

**Figure 1:**
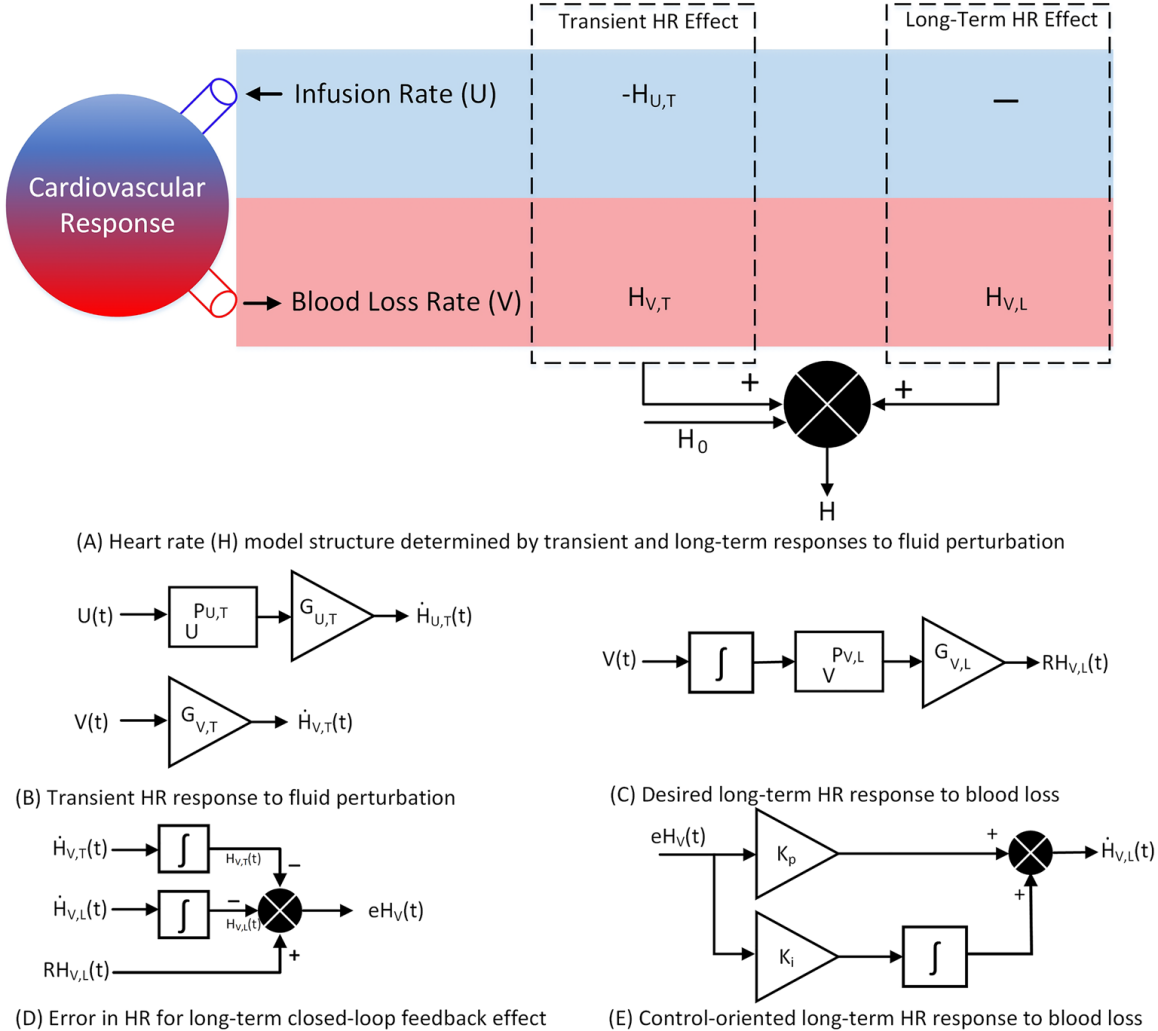
Model overview: (**A**) the model structure to map the hemorrhage and fluid infusion inputs to the heart rate response. The model consists of three parts. The first part maps the transient change in the heart rate due to hemorrhage, the second part maps the transient change in the heart rate due to fluid infusion, and the last part maps the long-term changes due to hemorrhage. (**B**) This illustrates how the model maps the transient changes in the heart rate due to hemorrhage and fluid infusion. (**C**) This shows the time-varying long-term target for the heart rate rise due to hemorrhage. (**D**), (**E**) This illustrates the control-oriented approach to modeling the long-term changes in the heart rate due to hemorrhage.

We developed a mathematical model of HR response to fluid perturbation that maps the hemorrhage and fluid infusion rates as the model’s inputs to the HR response, an important physiological variable during fluid perturbation. Physiologically, after a hemorrhagic shock, a rise in the HR response, known as tachycardia, is expected due to an increase in sympathetic nerve activity^28^. This transient increase due to severe hemorrhage could be followed by a long-term increase which is attributed to a loss of blood volume and the resulting decrease in arterial blood pressure^28^. Conversely, fluid infusion leads to a transient drop in the HR response, alleviating some effects of the hemorrhage^29^. Based on this physiological behavior of HR response, the model is designed to reflect both short-term or transient as well as long-term responses to fluid perturbation. The transient response is the instantaneous change in the HR due to hemorrhage and fluid infusion, while the long-term response refers to the change in the steady-state value of the HR response due to hemorrhage. As a result, the model is divided into three major parts, the transient response to hemorrhage, the transient response to fluid infusion, and the long-term response to hemorrhage, as shown in Fig. 1A. Figure 1B–E illustrates the parts of the model in detail.

For a given fluid loss with a rate of *V*(*t*) (l/m), considered as the sum of blood loss and urinary output over the time variable *t*, the model reflects an immediate increase in the HR response^28^. This transient increase, denoted as $H_{V,T}$ (beats per min, i.e., bpm), is modeled proportionally to the rate of loss via a constant gain of $G_{V,T}$ (1/lm) (see Fig. 1B):

1 $$\begin{aligned} H_{V,T}(t) = \int _{0}^{t} {\dot{H}}_{V,T}(l)dl = \int _{0}^{t} G_{V,T}V(l)dl \end{aligned}$$

Providing intravenous fluid *U*(*t*) (l/m) to compensate for the blood loss during hemorrhage somewhat alleviates the tachycardia response to the initial hemorrhage^29^. To account for this phenomenon, the change in HR response due to infusion, denoted as $H_{U,T}$ (bpm), is modeled through a nonlinear algebraic equation with a constant power term of $P_{U,T}$ (-) and a gain of $G_{U,T}$ (1/lm) (see Fig. 1B):

2 $$\begin{aligned} H_{U,T}(t) = \int _{0}^{t} {\dot{H}}_{U,T}(l)dl = \int _{0}^{t} G_{U,T}U(l)^{P_{U,T}}dl \end{aligned}$$

Lastly, there is a long-term effect on the HR response due to a severe loss in blood volume and the resulting drop in arterial blood pressure^28^. To incorporate this effect, the model utilizes a control-oriented modeling approach, in which a proportional-integral (PI) controller enforces the HR response to follow an expected long-term rising pattern due to the hemorrhage. The time-varying target for the steady-state HR response $RH_{V,L}$ (bpm) is defined through a nonlinear algebraic equation with a constant power term of $P_{V,L}$ (-) and a gain of $G_{V,L}$ (1/lm), which is integrated over the course of fluid perturbation, where a consistent tachycardia hemodynamic status is in effect (see Fig. 1C):

3 $$\begin{aligned} RH_{V,L}(t)=\int _{0}^{t} G_{V,L} V(l)^{P_{V,L}}dl \end{aligned}$$

The PI controller continuously evaluates the discrepancy between the target steady-state HR response Eq. (3) and simulated HR at each time and changes the HR response as needed to follow the target using the controller gains $K_{p}$ (1/m) and $K_{i}$ (1/m$^2$) (see Fig. 1D, E):

4 $$\begin{aligned}&H_{V,L}(t) = \int _{0}^{t} {\dot{H}}_{V,L}(l)dl = \int _{0}^{t} \left( K_{p} eH_{V}(l) +K_{i}\int _{0}^{l} eH_{V}(f)df\right) dl \end{aligned}$$

5 $$\begin{aligned}&H_{V}(t) = H_{V,T}(t)+H_{V,L}(t) \end{aligned}$$

6 $$\begin{aligned}&eH_{V}(t) = RH_{V,L}(t) - H_{V}(t) \end{aligned}$$

Finally, the model calculates the total change in HR due to fluid perturbation. This is achieved by combining the change in the HR induced by the hemorrhage as well as fluid infusion. The model then predicts the HR response by adding this change to the baseline HR, denoted as $H_0$ (bpm):

7 $$\begin{aligned} H(t) = H_0+H_{V,T}(t)+H_{V,L}(t)-H_{U,T}(t) = H_0+H_{V}(t)-H_{U,T}(t) \end{aligned}$$

### Parameter estimation

The model provides an estimate of the HR response at each discrete time $t_k$:

8 $$\begin{aligned} H_{obs} (t_k) = H(t_k|\Theta ) + \varepsilon (t_k) \end{aligned}$$

where $H(t_k)$ (bpm) represents the HR estimation provided by the model and $H_{obs} (t_k)$ represents the observed experimental HR response to fluid perturbation. $\Theta =\{G_{V,T},G_{U,T},G_{V,L},P_{V,L},P_{U,T},K_{p},K_{i},H_{0}\}$ represents the set of parameters that build the model. $\varepsilon (t_k)$ represents the error between the output of the model and the observed HR value at each time. We assume that the error has a normal distribution with a mean of 0 and a variance of $\sigma ^2$:

9 $$\begin{aligned} N(\varepsilon ;0,\sigma ^2) = \frac{1}{\sqrt{2\pi \sigma ^2}} exp\left\{ \frac{-1}{2\sigma ^2} (H_{obs} (t_k)-H(t_k|\Theta ))^2\right\} \end{aligned}$$

Thus, assuming the errors at different times are independent, the likelihood of parameters $\Theta$ and $\sigma$ given the observed data, is:

10 $$\begin{aligned}&L(\Theta ,\sigma |H_{obs}(t_k)) = \prod _{k=1}^{K} N(H_{obs} (t_k);H(t_k|\Theta ,\sigma ^2))=(2\pi \sigma ^2)^{-K/2} exp\left\{ \frac{-1}{2\sigma ^2} (H_{obs} (t_k)\right. \nonumber \\&\quad \left. -H(t_k|\Theta ))^T (H_{obs} (t_k)-H(t_k|\Theta )) \right\} \end{aligned}$$

By taking the logarithm of the above equation, the log-likelihood is defined by the following equation:

11 $$\begin{aligned} L^*(\Theta ,\sigma ) = \frac{-K}{2} ln(2\pi ) - \frac{K}{2} ln(\sigma ^2) -\frac{1}{2\sigma ^2} (H_{obs} (t_k)-H(t_k|\Theta ))^T (H_{obs} (t_k)-H(t_k|\Theta )) \end{aligned}$$

To fit the model to the observed HR and identify the optimum model parameters $\Theta ^*$ and $\sigma ^*$, the following optimization problem is defined:

12 $$\begin{aligned} \mu = \left\{ \Theta ^*,\sigma ^* \right\} = \underset{\mu }{arg min}(-L^*(\Theta ,\sigma ) + 2\gamma \left\| \Theta \right\| _{2}) \end{aligned}$$

where $\gamma$ refers to the penalty term applied and $\left\| . \right\| _{2}$ is a $L_2$ norm operator on the model parameters to reduce the chance of over-fitting. This function minimizes the negative log-likelihood to identify the combination of parameters with the minimum cost value.

### Experimental data

A study was conducted on 16 sheep. The data collection protocol was approved by the Institutional Animal Care and Use Committee (IACUC) at the University of Texas Medical Branch. All experiments were performed in accordance with relevant guidelines and regulations. The experimental protocol is described in detail elsewhere^30^, which is in accordance with ARRIVE guidelines. The data from this study were used to train and evaluate the mathematical model of HR response to fluid perturbation. All 16 sheep were given Lactated Ringer’s solution (LR) during fluid infusion. Amongst the 16, 5 sheep were also given Hextend (Hex) in a separate study. For these 5 sheep, the two studies were conducted 5 days apart. Each animal study lasted for 180 min. Before the beginning of each study, baseline data was collected from the subjects. At the start of the study, a 25 ml/kg hemorrhagic shock was induced in the subjects which lasted for 15 min. At 30 min, fluid infusion was started which continued till the end of the experiment. This infusion was performed for resuscitation with a target mean arterial pressure of 90 mmHg. At 50 and 70 min marks, two small 5 ml/kg hemorrhagic shocks were induced, each lasted for 5 min.

In addition to these subjects, a group of 6 separate sheep underwent the same protocol for hemorrhage, while at 30 min, fluid infusion was started by providing LR till the target mean arterial pressure of 65 mmHg is achieved. This dataset, called the independent validation dataset, is used to further validate the model. For more details about all data used in this research, refer to^30^.

### Model complexity optimization

Including the baseline HR, the developed model so far consists of 8 parameters in total. Analysis was done to ensure an adequate trade-off between the model complexity and its fitting performance. To achieve this, a global sensitivity analysis was performed based on the Morris method^31–33^. This method calculates the elementary effect for each parameter, which is defined as:

13 $$\begin{aligned} EE_{i}(\Theta ) = \frac{H(\theta _1,\ldots ,\theta _i+\Delta ,\theta _{i+1},\ldots \theta _k)-H(\Theta ))}{\Delta } \end{aligned}$$

**Figure 2:**
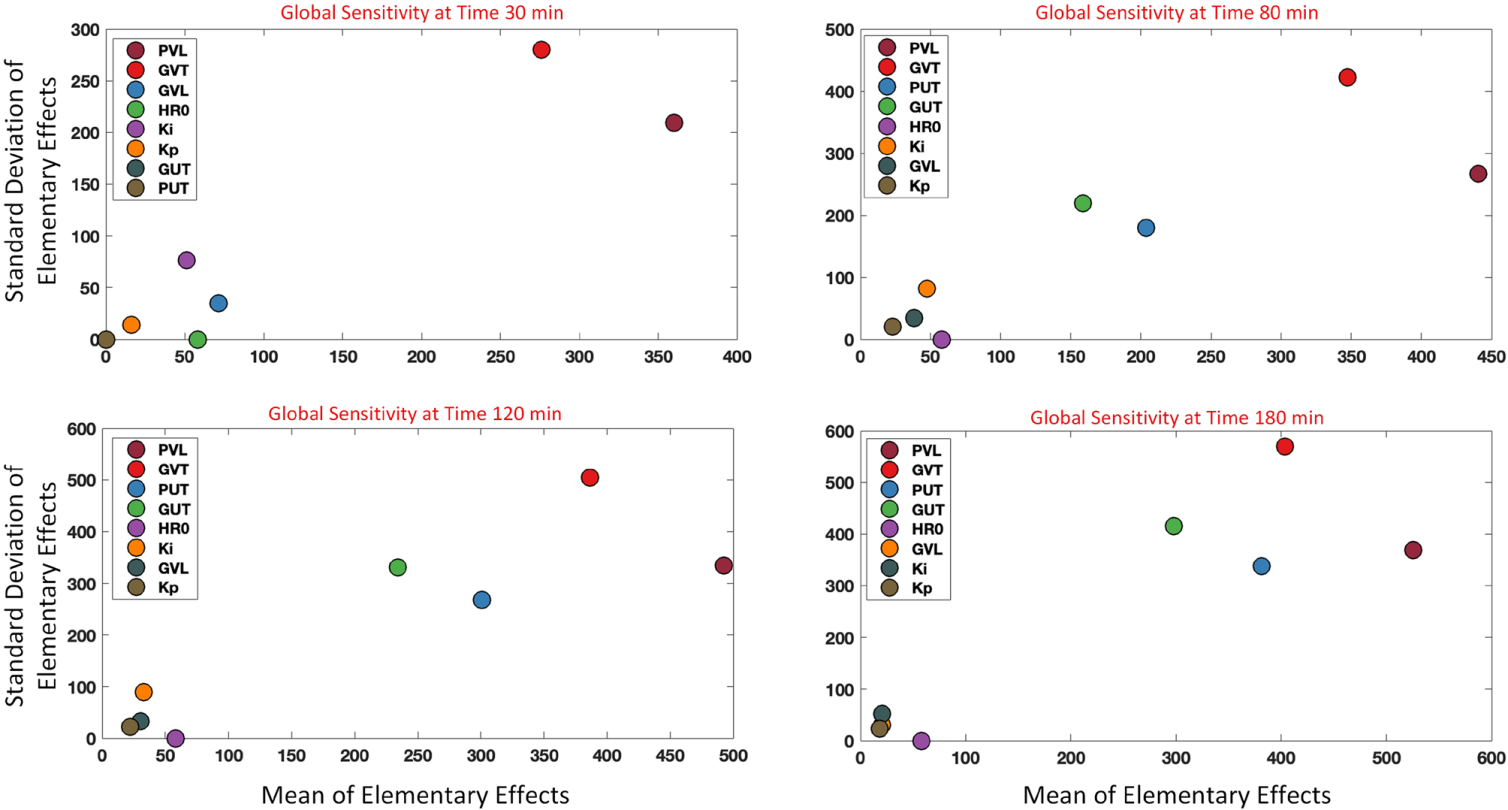
Sensitivity analysis using the Morris method for one representative subject. Analysis is done at 4 time points; 30 min, 80 min, 120 min, and 180 min. Mean and standard deviation of the Elementary Effect are plotted. A higher mean indicates that the parameter has a higher influence on the output of the model, while a higher standard deviation indicates higher interaction of the parameter with other parameters.

Here $\theta _i$ is the ith parameter and $\Delta$ represents the change in that parameter. A number of elementary effects are estimated for each $EE_{i}$ by randomly selecting $\Theta$ around the parameter being assessed. Next, to analyze the sensitivity ranking of each parameter, this method calculates the mean and the standard deviation of the elementary effect for each parameter across all selected points. A higher mean indicates that the parameter has a higher influence on the output of the model, while a higher standard deviation indicates higher interaction of the parameter with other parameters. The mean of the elementary effect was used to rank the sensitivity of parameters in each subject. The sensitivity analysis was performed on the data at 4 time points: 30 min (i.e., right before the infusion starts), 80 min (i.e., at the end of two small hemorrhages), 120 min (i.e., during the infusion), and 180 min (i.e., the end of the experiment when HR has potentially achieved its steady-state response). Figure 2 provides plots for the mean versus standard deviation for all the parameters at the 4 time points for a representative subject. It is noted that for consistency, we will present plots for the same representative subject in the “Model calibration assessment” and “Model validation” sections. This representative subject was selected to ensure sufficient inter-subject variability, in opposed to the situations with minimal response to blood loss and fluid infusion, to effectively challenge the model calibration and validation methods and also to ensure the subject is representative of the entire population with an average calibration and validation performance. Table 1 ranks the overall sensitivity of all parameters at the different time points. This was obtained by calculating the median ranking of each parameter across all subjects at each specific time. As seen from the table, $\hbox {K}_{\mathrm{p}}$ and $\hbox {K}_{\mathrm{i}}$ were the least sensitive parameters across all time points.

To create a simpler version of the model, any removal of the parameters should not largely degrade the fitting performance. In other words, an adequate trade-off between the model complexity and fitting performance should be obtained. This was investigated using the Akaike information criterion (AIC) defined as^34,35^:

14 $$\begin{aligned} AIC = 2P - 2L^{*} \end{aligned}$$

Where $L^{*}$ is the log-likelihood function defined in Eq. (11) and *P* is the number of model parameters. A lower AIC value across different number of parameters indicates a better trade-off. The AIC test was conducted on 3 versions of the model; a model with all the parameters, a model excluding $K_{p}$ only, and a model excluding $K_{i}$ only, where $K_{p}$ and $K_{i}$ were the least sensitive parameters (see Table 1). From the analysis, it was found that out of the 21 test cases, 14 had the lowest AIC using the model with all the parameters, and 7 had the lowest AIC using the model without $\hbox {K}_{\mathrm{p}}$. There were no cases where the model without $\hbox {K}_{\mathrm{i}}$ yielded the lowest AIC. Therefore the model with all 8 parameters was chosen as the final model.

**Table 1:** Sensitivity ranking.

Variable	${\hbox {T}_{30}}$	${\hbox {T}_{80}}$	${\hbox {T}_{120}}$	${\hbox {T}_{180}}$
$\hbox {G}_{\mathrm{V,T}}$	2	2	2	3
$\hbox {G}_{\mathrm{U,T}}$	7	4	4	4
$\hbox {K}_{\mathrm{p}}$	6	8	8	8
$\hbox {K}_{\mathrm{i}}$	5	6	7	7
$\hbox {HR}_{\mathrm{0}}$	4	5	5	5
$\hbox {P}_{\mathrm{U,T}}$	8	3	3	3
$\hbox {P}_{\mathrm{V,L}}$	1	1	1	1
$\hbox {G}_{\mathrm{V,L}}$	3	7	6	6

The table lists the ranking for each parameter across all four time points. At each time, the parameter with ranking 1 has the highest influence on the model’s output at that specific time.

### Model calibration assessment

The calibration performance for the final model was evaluated using the training dataset. The model was fitted using 21 test cases (see section “Experimental data”). The fitting performance in each subject was quantified by calculating the normalized root mean square error (NRMSE) between the model’s output and observed HR values: Here, *H* is the simulated HR after the model is calibrated to each subject. $H_{obs}$ is the experimental HR measurements. *N* is the total number of measurements in the subject.

15 $$\begin{aligned} NRMSE = \frac{\sqrt{\frac{\sum _{k=1}^{N}(H_{obs}(t_k)-H(t_k))^{2}}{N}}}{\overline{H_{obs}}} \end{aligned}$$

A t-test was also performed between the two groups receiving LR and HEX solutions to examine the differences in the models created under different resuscitation solutions.

## Model validation

With the finalized model structure and verified calibration performance against the training dataset, it is important to assess the model’s ability to predict HR response under normal and worst-case conditions. In this section, we propose three approaches for the assessment of predictive capability performance: subject-specific validation, cohort leave-one-out cross-validation, and cohort validation using the independent validation dataset. The first test assesses each subject’s model against data from the same subject that were not used in model calibration. The other two tests evaluate virtual cohorts, not individual models. These two tests examine whether a generated cohort adequately captures the range of possible responses while penalizing a cohort that excessively deviates from the observed responses.

### Subject specific assessment of predictive capability performance

Subject-specific predictive analysis examines the predictive capability performance of the model against conditions that were not present when the model was calibrated to an individual subject. A model can perform well on the data it was calibrated to, but poorly on new data even from the same subject, and thus, may not be able to generate simulations under the normal and worst-case conditions given its poor predictive capability performance. The model developed in this work should be able to adequately replicate HR response to input and boundary conditions that were not available when calibrating the model parameters.

To assess the subject-specific predictive capability performance, we calibrate the model to a portion of the data, and the remaining data is left for the prediction performance assessment. The assessment was performed for the following two portions of data in each individual: (1) the transient response, where the predictive performance was assessed in presence of both hemorrhage and infusion. In this analysis, the data between 45 and 80 min time range were used to examine the predictive performance while a sub-sample of the remaining data was used for model calibration. During 45–80 min, the amount of fluid perturbation is high, and thus, analyzing the corresponding model’s performance accounts for the condition under which large transient perturbations can occur during the fluid resuscitation; and (2) the steady-state response at the end of each experiment, i.e., the last 30 min, where the subject is under mostly steady fluid infusion. During the steady-state phase of the experiment, the amount of fluid perturbation is low with no presence of hemorrhage. Examining the model’s predictive performance against this portion of data allows us to analyze the performance of the model during a relatively stable condition. In this analysis, the data between 150 and 180 min time range were used to examine the predictive performance while a sub-sample of the remaining data was used for model calibration.

To quantify the model’s predictive capability performance, a bootstrapping analysis was applied. The model was calibrated 100 times to a 75% randomly selected sub-sample of the training data, i.e., data within 0–45 min and 80–180 min for transient response analysis and data within 0–150 min for steady-state response analysis^15^. Only 75% of the training data was used to avoid large uncertainty in parameter estimation and keep the number of experimental measurements about 3–4 times as large as the number of model parameters. The performance for these models was studied by analyzing the output of the model during the testing portion of data, i.e., 45–80 for the transient and 150–180 min for the steady-state response analysis. NRMSE was calculated between the predicted response of the 100 calibrated simulations and the observed values using Eq. (15).

### Leave-one-out assessment of predictive capability performance

A leave-one-out cross-validation analysis was performed to assess the predictive capability performance of the model against a subject not used for model calibration. A good predictive performance can ensure the model’s capability in replicating the normal and worst-case conditions during a fluid perturbation. The bleeding and infusion patterns from the left-out subject were considered as input to the model. The other 20 subjects were used to tune the model parameters for generating virtual patient cohorts. The validation was performed on all 21 subjects. Two methods were introduced to generate the virtual patient cohorts; the compartment method and the uniform distribution method. See section “Virtual cohort development” for details. To quantify the performance of generated virtual patients, a 95th percentile envelope was computed based on all simulated subjects, which covers the 2.5th–97.5th percentile of the prediction forecasts at each time point. The prediction envelope gives a representation of the most likely outputs of the model for the given input pattern.

After extracting the prediction envelope, a normalized interval score (NIS) is calculated at each time point. NIS is a modified version of the interval score developed by Gneiting and Raftery^36^. The interval score is normalized using the observed HR response for a more meaningful evaluation approach. This score rewards a narrow prediction envelope and applies a penalty if the prediction envelope does not contain the observed HR value. NIS is calculated using the following equation:

16 $$\begin{aligned} NIS(t_k) = {\left\{ \begin{array}{ll} \frac{(H_u (t_k)-H_l (t_k))+\frac{2}{\alpha }(H_l (t_k)-H_{obs} (t_k))}{H_{obs} (t_k)} &{} H_{obs} (t_k)< H_l (t_k)\\ \frac{(H_u (t_k)-H_l (t_k))+\frac{2}{\alpha }(H_{obs} (t_k)-H_u (t_k))}{H_{obs} (t_k)} &{} H_u (t_k) < H_{obs} (t_k)\\ \frac{H_u (t_k)-H_l (t_k)}{H_{obs} (t_k)} &{} Otherwise \end{array}\right. } \end{aligned}$$

Here $H_u (t_k)$ and $H_l (t_k)$ are the upper and lower bound of the prediction envelope.

**Figure 3:**
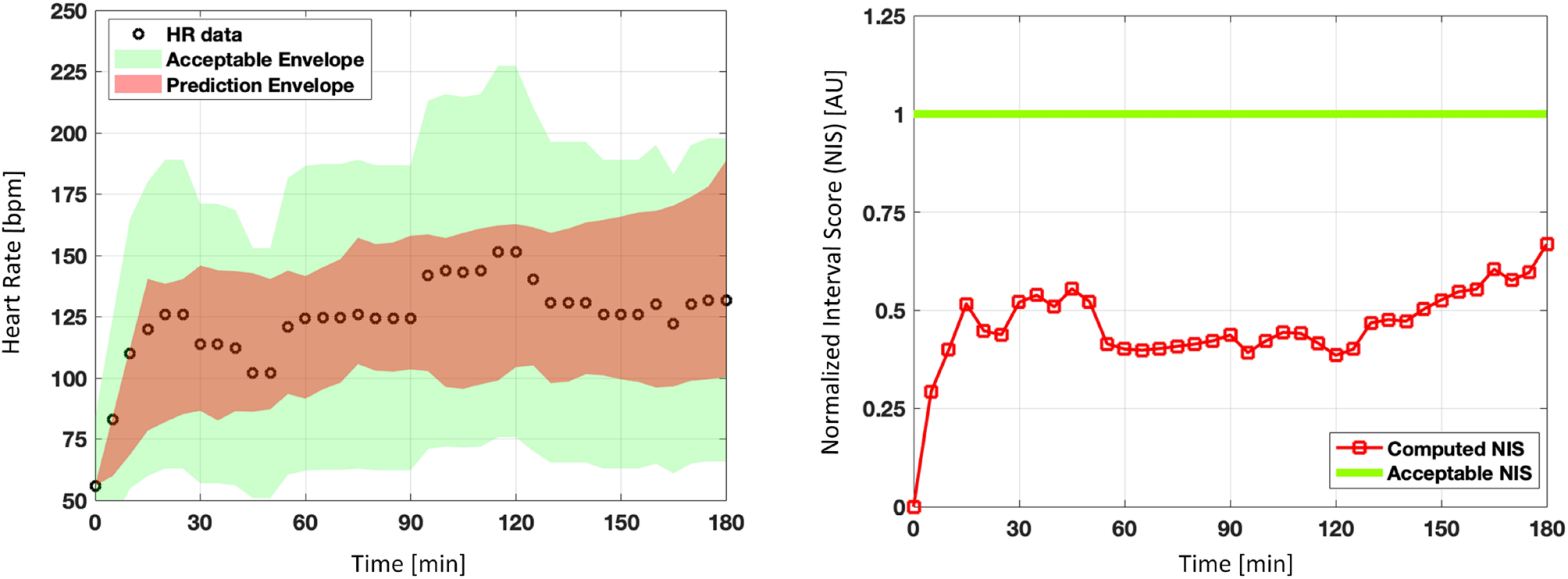
A representative assessment of predictive capability performance with acceptable NIS of 1. An acceptable NIS is defined that allows a maximum permissible deviation of the predicted heart rate value from the observed heart rate value ($\pm 50\%$ in the left figure). An acceptable NIS threshold of 1 is shown in the right figure. The generated envelope is acceptable if the computed NIS is equal or less than the acceptable NIS threshold.

To evaluate the performance, the calculated NIS was compared against an acceptable NIS. An acceptable NIS defines an envelope around the observed data that allows for a known amount of deviation. For example, if the acceptable NIS is 1 then the prediction value is allowed to deviate by $\pm 50\%$ of the observed value. Figure 3 illustrates the acceptable range definition. The plot on the left shows the prediction envelope and the acceptable envelope overlaid on the observed data. This plot shows the envelope for the acceptable NIS = 1. In this example, since the prediction envelope was narrower than the acceptable envelope, the NIS for the prediction envelope is lower than the acceptable NIS (see the plot on the right). Therefore, for an acceptable predictive capability performance, the calculated NIS should be statistically lower than the acceptable NIS.

**Figure 4:**
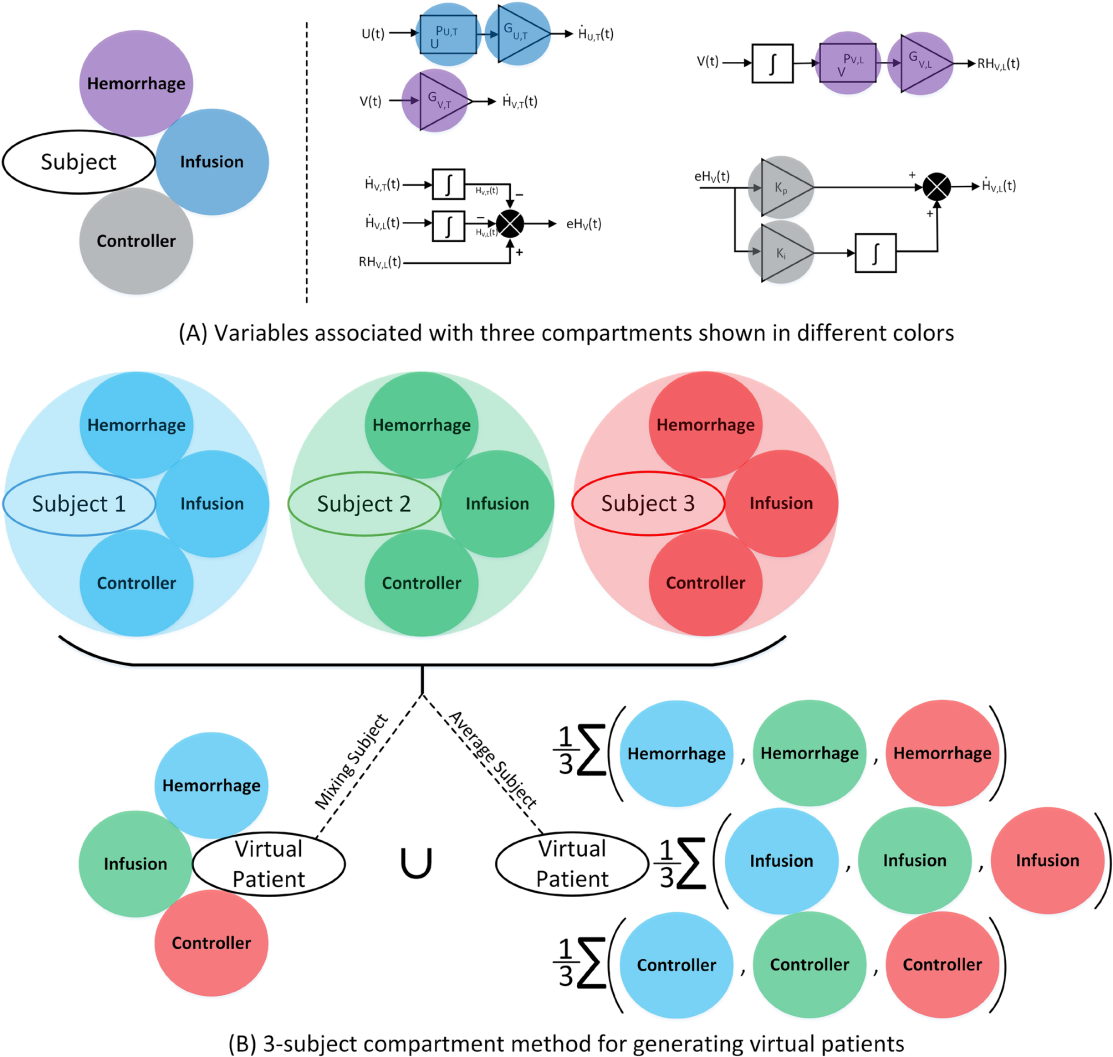
Compartment method of cohort generation: (**A**) the model parameters are divided into three compartments. These are (1) parameters that map the heart rate changes due to hemorrhage, (2) parameters that map the heart rate changes due to fluid infusion, and (3) parameters that define the controller. (**B**) The virtual subject is generated by (1) mixing the compartments from three different subjects and (2) by taking the average of the compartments from the three subjects.

### Assessment of predictive capability performance using an independent validation dataset

Lastly, the predictive capability performance of the model was tested using the independent validation dataset. In this method, hemorrhage and infusion patterns from 6 completely new subjects were used to test the model’s forecast. This validation set was collected using a different experimental protocol (see the “Methods” section). Therefore, this analysis also allows us to examine the use of the model in the worst-case conditions and physiological states that are possibly far from those used for model calibration. The model parameters used for virtual cohort generation were obtained from all 21 subjects in the calibration dataset. In addition, both the compartment and the uniform distribution methods were used to generate the virtual cohorts. In this procedure, similar to the leave-one-out approach, predictive capability performance was quantified by extracting the prediction envelope and calculating the NIS.

### Virtual cohort development

In this paper, we used a novel compartment method as well as a uniform distribution method to generate virtual subjects. This subsection illustrates the details of these cohort generation tools.

*Compartment cohort generation method* We developed a novel compartment cohort generation tool. The model parameters are divided into three compartments; the parameters associated with hemorrhage, fluid infusion, and lastly, the controller. Figure 4A illustrates these three compartments and their associated parameters. This method can be easily generalized to any number of compartments, depending on a mathematical model’s structure. When creating a virtual subject, this method selects 3 random subjects across the entire *n* calibration dataset ($n=20$ for the leave-one-out cross-validation and $n=21$ for validation using the independent dataset). Then, the virtual subject is built by selecting one compartment from each subject. For example, for subjects *i*, *j*, and *k*, the virtual subject can be constructed using the parameters from subject *i*’s hemorrhage compartment, subject *j*’s infusion compartment, and subject *k*’s controller compartment, or any combination of them, named mixing method (see Fig. 4B). Apart from that, another virtual subject can be generated by taking the average of parameters associated with each compartment between the three subjects. With the entire *n* subjects in the calibration dataset and using the 3-subject mixing method, $n^3$ virtual patients can be simulated. Similarly, using the 3-subject average method, another $n^3$ virtual patients can be simulated, leading to 2$n^3$ virtual subjects in total, that is, 16,000 and 18,522 subject for the leave-one-out cross validation and validation using the independent dataset, respectively. The virtual subject created using the compartment method has a high chance of being physiological since the combination of parameters comes from the actual subjects used for model calibration and not random parameter values that are chosen independently from the rest of the parameters. For more details about the three-subject compartment method, see Fig. 4B.

*Uniform distribution cohort generation method* Uniform distributions were created for each model parameter. The lower and upper bounds for the distribution of a parameter are respectively the minimum and maximum values for that parameter across the entire *n* subjects used for model calibration. For each generated virtual subject, the value of the parameter is randomly sampled from the uniform distribution. Similar to the compartment method, 16,000 and 18,522 subjects are generated for each input profile in the leave-one-out cross validation and validation using the independent dataset, respectively.

When creating the virtual subject using both compartment and uniform distribution methods, some simulations may not yield HR values that are within the physiological range, defined here as a range of 40–250 bpm. These types of simulations are discarded and labeled as non-physiological virtual subjects. The remaining simulations that are within the physiological range are labeled as physiological virtual subjects. Furthermore, some of these physiological subjects may have a pattern that is dissimilar to the pattern exhibited by the actual subject. While such simulations can represent a valid subject, they cannot be fairly used to compare with the actual subject. Therefore, only simulated subjects with the selected NRMSE threshold of $\le$ 20% were considered relevant to the test subject and included in the envelope when compared to the observed HR response. This is consistent with prior studies in defining behavioral samples for model evaluation using likelihood measures that evaluates the fitness of the model to the observed data, e.g., generalized likelihood uncertainty estimation (GLUE)^37^. This NRMSE threshold can be adjusted for other applications or physiological variables.

## Results

In this section, we present the calibration performance of the model. We also provide the results for subject-specific prediction performance. Finally, we show the results for the predictive capability performance of the leave-one-out cross-validation and the independent validation dataset using two methods of virtual cohort generation.

### Model calibration assessment

**Figure 5:**
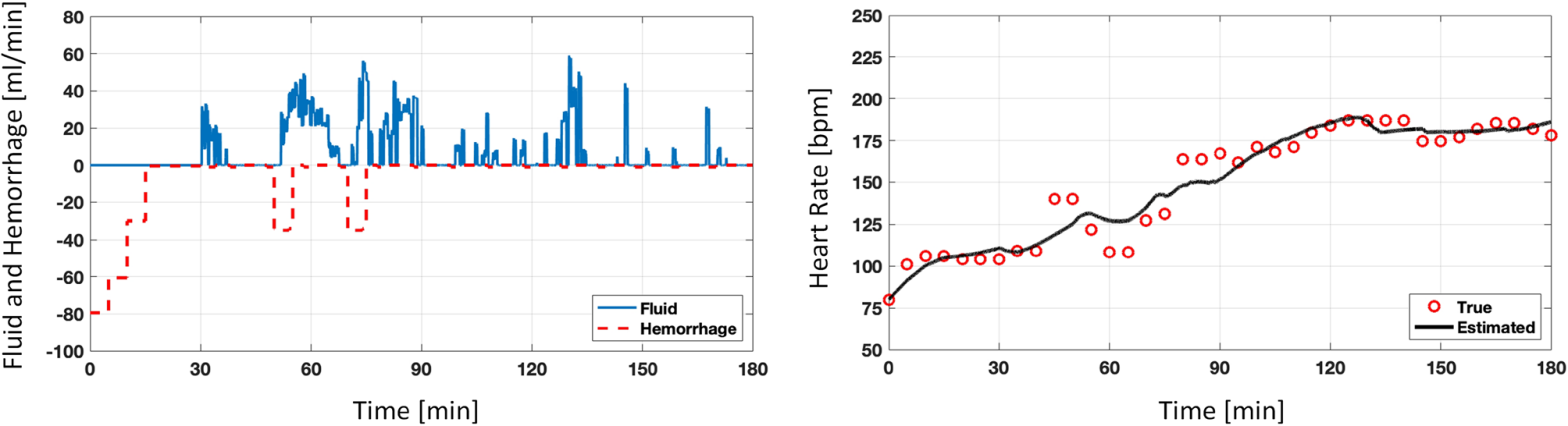
Model calibration performance: This figure illustrates the model estimated heart rate to fluid perturbation in one representative subject. The graph on the left shows the hemorrhage (red) and infusion (blue) patterns. The graph on the right shows the subject’s observed heart rate (red) and the model’s estimated heart rate response (black).

Figure 5 illustrates the model fitting performance for one representative subject. The plot on the right shows the model estimated HR, which closely tracks the observed data. Table 2 lists the median and interquartile range values for the parameters calibrated to the subjects receiving LR and HEX fluid solutions. The t-test results show that none of the parameters differed significantly between the models built under LR versus HEX solutions (paired t-test, *p* > 0.05). NRMSE was calculated between the observed and estimated HR to quantify the performance (see Table 3). Across all 21 subjects used for calibration, the average NRMSE was found to be $7.41 \pm 2.8 \%$, indicating that the model was able to estimate the observed HR response to fluid perturbation with a small error.

**Table 2:** The table lists the median values of all parameters in the model across 21 calibration datasets.

	Hex	LR	All
$\hbox {G}_{\mathrm{U,T}}$	2.1 (1.5, 102.1)	17.3 (0.96, 176.2)	16.9 (1.2, 138.8)
$\hbox {G}_{\mathrm{V,T}}$	13.5 (4.8, 67.9)	20.8 (5.2, 73.7)	20.2 (4.8, 71.8)
$\hbox {P}_{\mathrm{U,T}}$	0.5 (0.1, 0.9)	0.8 (0.04, 1.4)	0.7 (0.1, 1.4)
$\hbox {P}_{\mathrm{V,L}}$	0.1 ($8 \times 10^{-4}$, 0.7)	0.2 (0.03, 0.6)	0.2 ($8 \times 10^{-4}$,0.6)
$\hbox {G}_{\mathrm{V,L}}$	2.9 (1.4, 83.7)	6.2 (1.9, 17.5)	5.7 (1.8, 17.4)
$\hbox {K}_{\mathrm{p}}$	$5 \times 10^{-6}$ ($3 \times 10^{-6}$, 0.01)	$1 \times 10^{-3}$ ($7 \times 10^{-7}$, 0.01)	$3 \times 10^{-5}$ ($7 \times 10^{-7}$, 0.01)
$\hbox {K}_{\mathrm{i}}$	$4 \times 10^{-3}$ ($1 \times 10^{-3}$, $7 \times 10^{-3}$)	$2 \times 10^{-3}$ ($1 \times 10^{-3}$, $1 \times 10^{-2}$)	$2 \times 10^{-3}$ ($1 \times 10^{-3}$,$8 \times 10^{-3}$)
$\hbox {H}_{\mathrm{0}}$	79 (60.3, 81.1)	78.5 (70.0, 90.5)	79 (67.5, 84.9)

The values in parentheses list the interquartile ranges of the parameter. Values were separated for subjects receiving the Hextend (Hex) solution and those receiving the Lactated Ringer’s (LR) solution. The table also lists the values for all subjects together.

**Table 3:** This table shows the average minimum NRMSE and average NRMSE between estimated or predicted and observed heart rate using the model calibration and subject-specific validation methods.

	Average minimum NRMSE	Average NRMSE
Model calibration	–	7.41 ± 2.8
Subject specific prediction: transient	7.63 ± 3.29	11.83 ± 7.37
Subject specific prediction: steady state	7.66 ± 9.89	16.27 ± 12.93

The minimum NRMSE quantifies the performance of the prediction that most closely replicates the observed heart rate.

### Subject specific predictive capability performance

**Figure 6:**
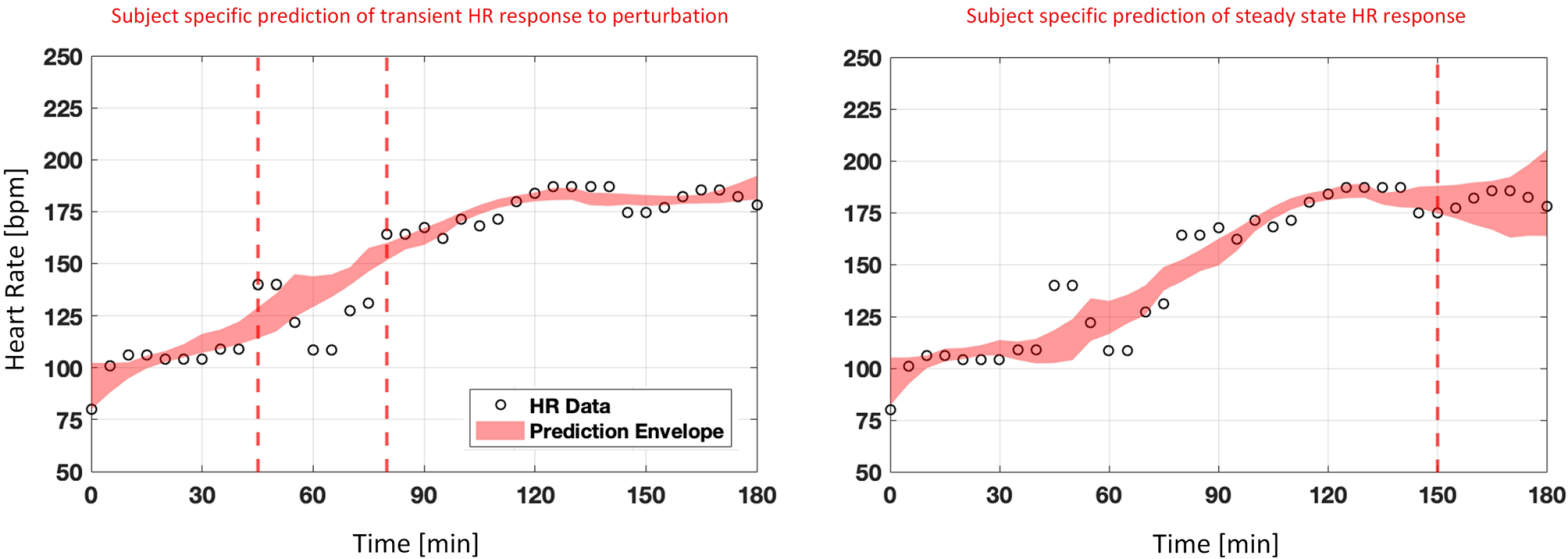
Subject specific prediction assessment under two scenarios. (**A**) Left: prediction envelope for the transient response (45–80 min) by fitting the model to a sub-sample of heart rate measurement between 0–45 and 80–180 min. (**B**) Right: prediction envelope for the steady-state response (150–180 min) by fitting the model to a sub-sample of heart rate measurements between 0 and 150 min. Refer to Fig. 5 for the hemorrhage and fluid infusion profile.

The assessment analysis was performed during the transient and steady-state HR response to fluid perturbation. Figure 6 illustrates the model performance in one representative subject for subject-specific prediction of HR. The plots show the prediction envelope obtained from bootstrapping overlaid on the observed HR. The area between the vertical lines is the portion of the data that was not considered during model calibration.

To quantify the performance, all 100 bootstrapped predictions were compared to the observed HR. Table 3 lists the overall NRMSE between the two, where the minimum NRMSE indicates the best bootstrapped prediction that the model was able to generate. The average minimum NRMSE for subject-specific prediction during transient and steady-state was $7.63 \pm 3.29\%$ and $7.66 \pm 9.89\%$, respectively. This indicates that the model was able to adequately predict at least one subject-specific HR for input and boundary conditions that were not present during calibration. In addition, the average NRMSE was calculated for each subject, which turned out to be $11.83 \pm 7.37\%$ and $16.27 \pm 12.93\%$ for transient and steady-state subject-specific prediction, respectively.

### Leave-one-out assessment of predictive capability performance

**Figure 7:**
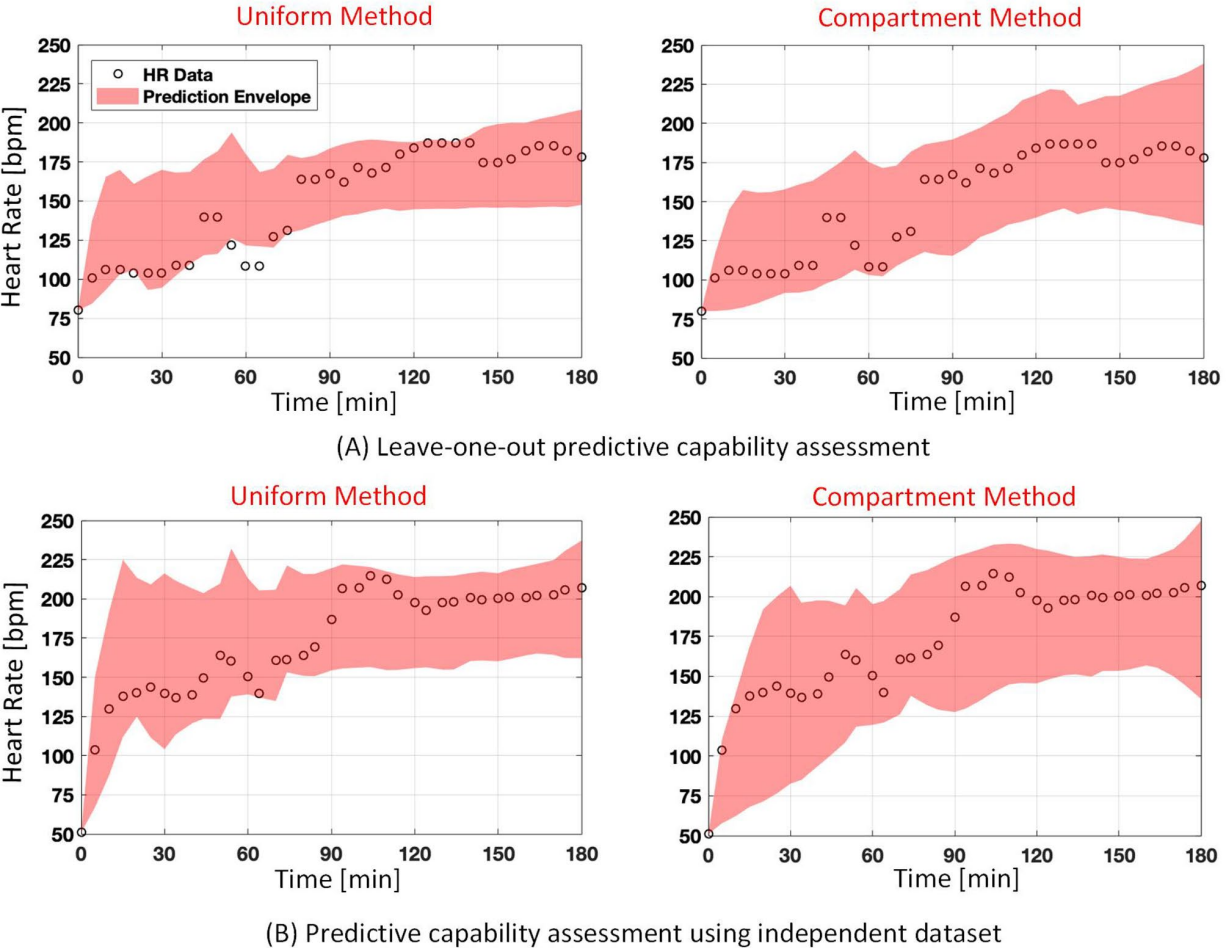
Predictive capability assessment for a fully virtual subject. (**A**) The plots depict the performance using the leave-one-out approach with the uniform method (left) and the compartment method (right) of cohort generation. (**B**) The plots depict the performance using the independent dataset approach with the uniform method (left) and the compartment method (right) of cohort generation.

The performance of the model in predicting a fully virtual subject was first examined using the leave-one-out cross-validation approach. Both uniform distribution and compartment methods were used to create virtual subjects. Figure 7A illustrates the 95th percentile predictive envelop in one representative subject. The figure shows performance using both the uniform distribution method (left) and the 3-subject compartment one (right). To quantify performance, the virtual subject that most closely resembled the actual subject was identified. This was done by selecting the virtual subject with the minimum NRMSE when compared to the observed data. Table 4 lists the average minimum NRMSE across all subjects. Using the leave-one-out approach, the average minimum NRMSE for the uniform distribution method and compartment method was respectively $12.91 \pm 5.34\%$ and $9.56 \pm 3.15\%$, indicating the compartment method was able to generate virtual subjects that were closer to the actual subjects than the uniform method when same number of samples are generated in each method. This was also confirmed by the analysis of the number of virtual subjects listed in Table 5 for both methods of cohort generation. The table lists the number of subjects out of a total of 16000 that were within the physiological range and the number of subjects who met the NRMSE threshold. As can be seen from the table, the number of virtual subjects within the physiological range as well as the number of subjects meeting the NRMSE threshold were significantly higher in the compartment method using the leave-one-out approach (paired t-test, within the physiological range: *p* = $1 \times 10^{-14}$; meeting the NRMSE threshold: *p* = $2 \times 10^{-5}$). In some cases, none of the virtual subjects were able to meet the NRMSE threshold using the uniform distribution method.

We also quantified the predictive performance of the model by calculating the NIS shown in Eq. (16). The calculated NIS was then compared against the acceptable NIS levels, which defines an envelope around the observed data that allows for a known amount of deviation. The cohort generation was analyzed with 4 acceptable NIS levels; 0.25, 0.5, 0.75, and 1 which allowed for a deviation of $\pm 12.5\%, \pm 25\%, \pm 37.5\%$, and $\pm 50\%$, respectively. To analyze whether the model performs better than the acceptable threshold, a paired t-test between the calculated NIS and the acceptable NIS was performed for each subject. Table 6 shows the percentage of subjects for whom the calculated NIS was statistically lower than the acceptable NIS (paired t-test, $p <0.05$). It was observed that with a more stringent acceptable NIS (0.25), there were very few subjects with a lower NIS. As the acceptable NIS becomes less strict, the percentage of subjects for whom the calculated NIS was lower increases. Lastly, across all acceptable NIS levels and compared to the uniform distribution method, compartment method showed a superior NIS performance.

**Table 4:** Average minimum NRMSE for model validation.

	Uniform distribution method	Compartment method
Leave-one-out	12.91 ± 5.34	9.56 ± 3.15
Independent set	13.65 ± 5.32	11.1 ± 1.22

The predicted heart rate values were compared to the observed heart rate values. The minimum NRMSE quantifies the performance of the prediction that most closely approximates the observed heart rate.

**Table 5:** Average number of virtual subjects for both uniform distribution method and the compartment method for model validation.

	Uniform distribution method		Compartment method	
	Physiological	NRMSE $\le$ 20%	Physiological	NRMSE $\le$ 20%
Leave-one-out	1120 ± 743	110 ± 141	5956 ± 1772	829 ± 686
Independent set	1685 ± 747	247 ± 307	7737 ± 568	942 ± 1062

The table lists the number of simulations that were within the physiological range as well as the number of simulations that satisfy the NRMSE criteria.

**Table 6:** Percentage of subjects satisfying the acceptable criteria with the normalized interval score equal or less than the acceptable NIS threshold.

	Uniform cohort method				Compartment method			
Acceptable range	0.25	0.5	0.75	1	0.25	0.5	0.75	1
Leave one out	4.76%	66.66%	76.19%	76.19%	0%	66.66%	90.47%	95.23%
Independent set	0%	66.66%	66.66%	66.66%	0%	50%	83.33%	100%

### Assessment of predictive capability performance using independent validation dataset

Similar to the leave-one-out approach, the model predictive performance in creating a fully virtual subject was examined using an independent validation dataset. Virtual subjects were created using both uniform distribution and compartment methods. Furthermore, only virtual subjects that showed a similar HR pattern as the actual subject (NRMSE $\le 20\%$) were used for further analysis. Figure 7B illustrates the predictive capability performance in one representative subject within the validation dataset. The figure shows the performance using both uniform distribution and compartment methods. Table 4 lists the average minimum NRMSE for both uniform distribution and compartment methods across all validation subjects, giving a measure of the virtual subject that most closely resembled the observed data. The minimum NRMSE averaged across all validation subjects for uniform distribution and compartment methods were $13.65 \pm 5.32\%$ and $11.1 \pm 1.22\%$, respectively. This indicates that the compartment method was able to generate virtual subjects that were closer to the actual subjects than the uniform distribution method. This was also confirmed by analyzing the number of subjects that were within the physiological range and met the NRMSE threshold (Table 5). As can be seen from the table, the number of virtual subjects within the physiological range was significantly higher in the compartment method (paired t-test, *p* = $2 \times 10^{-5}$). The number of subjects meeting the NRMSE threshold was on average higher in the compartment method, but the difference was not statistically significant (paired t-test, *p* = 0.11). The NIS for the prediction envelope was also calculated and compared against the acceptable NIS. Table 6 lists the results of this comparison. Similar to the leave-one-out approach, the percentage of subjects for whom the calculated NIS was lower increases as the acceptable NIS becomes less stringent. Furthermore, same as the leave-one-out approach, overall across all acceptable NIS levels, the compartment method showed a superior NIS performance, showing the importance of virtual cohort generation tool selection when evaluating a mathematical model.

## Discussion

This research intends to develop a mathematical model of HR response to be used as part of a larger testing platform for evaluating PCLC medical devices for fluid resuscitation. To achieve this goal, a model was developed that maps the rate of bleeding and infusion to the HR changes due to hemorrhage and fluid infusion. The proposed model decomposes the effects of the fluid perturbation into the transient and the long-term responses: the transient changes due to hemorrhage and fluid infusion, while the long-term changes attributed to hemorrhage only. The developed model contained 8 parameters including the baseline HR. The model employs a control-theoretic approach to effectively abstract the extremely sophisticated regulatory mechanisms for HR in the body in order to replicate the long-term steady-state response.

Efforts were made to simplify the model. In particular, the sensitivity of each parameter was examined using the global sensitivity Morris method. It was found that among the parameters, $\hbox {K}_{\mathrm{p}}$ and $\hbox {K}_{\mathrm{i}}$ were the least sensitive parameters. An investigation was done to verify whether removing any of these parameters adversely affects the performance of the model. It was observed that removing either parameter degraded the calibration performance, which was evidenced by the AIC. This concluded the model with all 8 parameters as the final model. The model was also evaluated for its calibration performance. The model was fitted to 21 sheep subjects. The averaged NRMSE across all subjects turned out to be $7.41 \pm 2.8\%$, indicating that the model was able to reasonably replicate the HR response to fluid perturbation with a small error.

Different rounds of analysis were performed to assess the model’s predictive capability performance. First, an analysis was performed to evaluate the model’s ability to predict HR patterns under the input and boundary conditions to which the model was not calibrated in an individual subject. A bootstrapping was performed and the model was calibrated to a sub-sample of an individual’s calibration data 100 times. The calibrated models were used to forecast the remaining testing data during the transient (45–80 min) and steady-state (150–180 min) portion of the data. The minimum NRMSE between the observed and predicted HR response within the time range of interest averaged across all 21 subjects was $7.63 \pm 3.29\%$ and $7.66 \pm 9.89\%$ for transient and steady-state responses, respectively. Compared to the model calibration performance above, results show that the model can lead to predictions similar to when a pure calibration is performed, indicating the high efficacy of the model in predicting subject-specific data. Moreover, the NRMSE across all 100 forecasts and averaged between all subjects were $11.83 \pm 7.37\%$ and $16.27 \pm 12.93\%$, for the transient and steady-state responses, respectively. Calibrating the model to some sub-samples of subject-specific data could lead to forecasts that are not close enough to the observed data with a relatively larger confidence interval, leading to an error to be larger than the calibration results. All in all, the averaged NRMSE is within an acceptable range and provides further evidence of the model’s suitability in forecasting subject-specific HR response to fluid perturbation.

Second, an analysis was performed to evaluate the model’s ability to generate fully virtual subjects. This analysis was performed using two separate approaches: prediction assessment using leave-one-out cross-validation and independent validation dataset. In the leave-one-out approach, the virtual subject is tested using hemorrhage and infusion patterns in one subject while the model parameters were calibrated to the remaining 20 subjects. In the second method, the model was calibrated to all 21 subjects, while tested against independent data collected under a different experimental protocol. In both approaches, two methods of cohort generation were used: the uniform distribution method and the 3-subject compartment method. The virtual subject that best represents the data was identified by calculating the NRMSE between the observed and predicted data. Using the leave-one-out cross-validation, the averaged minimum NRMSE for the uniform distribution and the compartment methods was $12.91 \pm 5.34\%$ and $9.56 \pm 3.15\%$, respectively. Furthermore, with the independent validation dataset, the averaged minimum NRMSE for uniform distribution and compartment methods was $13.65 \pm 5.32\%$ and $11.1 \pm 1.22\%$, respectively. The averaged minimum NRMSE in both approaches is comparable to the results from pure calibration, indicating that the model was able to generate at least one virtual subject that adequately represents the observed data with a small error.

To quantify the overall model’s predictive capability performance in replicating the observed data, NIS was calculated over the 95th percentile confidence interval of the prediction envelop with relevant simulations (i.e., NRMSE $\le 20\%$) for each subject. The non-relevant virtual subjects with NRMSE$>20\%$ can still represent an actual subject, but may not be used to compare to the test subject. Although these virtual subjects were removed from the envelope to effectively evaluate the model predictive capability performance, they could still be included in the population of virtual subjects used for testing a PCLC medical device performance. The NIS was compared to different acceptable NIS thresholds. For the cohort generation tool to be considered viable, the calculated NIS should be equal or smaller than the acceptable threshold of interest. The results in Table 6 show that the number of tests that meet the NIS criteria increases as the acceptance criteria for both uniform distribution and compartment methods becomes less stringent. This can be observed with both the leave-one-out and independent validation dataset approaches. The results show that with the leave-one-out approach, 95.23% of the tests using the compartment method and 76.19% of the tests using the uniform method presented HR patterns that were within a deviation of 50% (NIS=1) from the patterns exhibited by the actual subject. A more stringent criterion of 37.5% was met by 90.47% and 76.19% of the tests using compartment and uniform distribution methods, respectively. Similar results can be observed with the independent dataset approach. The selected acceptable NIS threshold depends on the application and physiological variable, where given the large variability of HR due to the sensor noise as well and effects from autonomic nervous system, a deviation of 37.5% or 50% (0.75 or 1) around the observed data could be acceptable. These results support the validity of the model and the cohort generation tool to create virtual subjects that closely resemble actual subjects under fluid perturbation. These results also indicate that the compartment method was able to generate virtual subjects that more closely resemble the actual subjects at a higher rate. This was confirmed by analyzing the number of virtual subjects falling within the physiological range and the number of subjects meeting the NRMSE threshold listed in Table 5. As can be seen from the table, in both leave-one-out approach and the independent dataset, the compartment method was able to generate more viable virtual subjects than the uniform distribution method when the same number of samples were generated in each method. Additionally, in some cases, the uniform distribution method was unable to generate virtual subjects that met the NRMSE threshold. This improvement in the compartment method is likely due to its nature, where the virtual subjects created using the compartment method have a high chance of being physiological since the combination of parameters comes from the actual subjects derived from model calibration and not from random parameter values that are chosen independently from each other. Selecting random individual parameters within a specified range can lead to a combination of parameters with lower chance of being physiological or relevant to a test dataset. On the other hand, the uniform method covers the entire range of probable values for the parameters, and thus, it may generate valid rare subjects that their pattern are dissimilar to the data used in model calibration. The predictive capability performance includes mixed effects from the model and the virtual cohort generation tool, and thus, these results indicate the importance of an adequate virtual cohort generation tool when evaluating a model. Applying the novel compartment method and also the uniform distribution method separately ensured an appropriate separation between the effects from the model and those of virtual cohort generation tool on the predictive capability performance.

The analysis performed on this model used retrospective data and hemorrhage and infusion protocols experienced by an actual subject. An adequate assessment of the model predictive performance for these data required certain considerations, for example only virtual subjects with similar pattern to the actual subjects (NRMSE $\le 20\%$) were used for the analysis. This step was necessary and consistent with prior studies, as some virtual subjects may lead to dissimilar patterns of HR response. Those patterns can still represent valid virtual subjects but may not be used to fairly assess the predictive capability performance. Assessment of future PCLC devices does not require the use of an NRMSE threshold. However, due to the nonlinear nature of the model, some virtual subjects may exhibit oscillating responses that need to be removed. In our analysis, the NRMSE threshold was able to remove these subjects. Using prospective data, filters must be incorporated that remove these oscillating virtual subjects.

The uncertainty in the data was not explicitly considered in this study. We developed and validated a HR model that corresponds only to the physiological response, while the measured animal data may be contaminated by noise. Additional future work, including hardware-in-the-loop simulation, is needed to better understand the impact of uncertainty in the data and noise in the measurement on the HR model calibration, model validation and in silico assessment of PCLC medical devices for fluid resuscitation. Furthermore, given the simplicity of the presented model, the transient response (45–80 min) and its corresponding peaks due to the two small hemorrhage perturbations were not completely captured in some subjects (including the one presented in Fig. 6). Some additional work may be needed to further improve the model performance during boundary conditions with sharp transient perturbation.

## Conclusion

This paper presented and validated a control-oriented mathematical model to simulate the changes in the HR during hemorrhage and fluid infusion. The results support the validity of the developed model to be incorporated into future non-clinical simulated testing setups for PCLC devices for fluid resuscitation of patients with hemorrhage. Using the model, a virtual cohort of subjects can be created that can be used in place of actual subjects to evaluate these devices. Sensitivity and AIC analyses were performed to verify the adequacy of trade-off between the model complexity and calibration performance. A fitting performance analysis conducted on the model showed that the model in the majority of the subjects is able to map the changes in the HR due to fluid perturbation with a reasonably small error. The results also show that the model in most cases was able to predict subject-specific transient and steady-state HR responses to fluid perturbation with an acceptable error margin, verifying the suitability of the model in replicating HR under different boundary conditions and input profile. Finally, results on the model’s predictive capability performance show that the model was able to create fully virtual subjects that resemble an actual subject. It was shown that the method of virtual cohort generation directly impacts the predictive performance, an important consideration in evaluation of test methods that use a model to generate simulated populations. The compartment method of cohort generation performed better than the uniform method in replicating the validation dataset. However, it is possible for the uniform method to generate rare but valid patterns of HR response that are not observed in the calibration data.

## Data Availability

The mathematical model developed in this work as well as the validation procedures during the current study will be available from the corresponding author on reasonable request.
